# Simultaneous Laparoscopic Cholecystectomy and Endovascular Infrarenal Aortic Aneurysm Repair

**DOI:** 10.3389/fsurg.2021.659961

**Published:** 2021-06-14

**Authors:** Giulio Illuminati, Francesco G. Calio', Rocco Pasqua, Priscilla Nardi, Chiara Fratini, Paolo Urciuoli

**Affiliations:** ^1^Department of Surgical Sciences, University of Rome “La Sapienza”, Rome, Italy; ^2^Department of Vascular Surgery, Sant'Anna Hospital, Catanzaro, Italy

**Keywords:** infrarenal aortic aneurysm, cholelithiasis, EVAR, laparoscopic cholecystectomy, minimally invasive treatment

## Abstract

**Background/Aim:** With the increasing use of endovascular aneurysm repair (EVAR) and the availability of laparoscopic cholecystectomy (LC) for treating abdominal aortic aneurysms (AAA) and cholelithiasis, respectively, the association between these elective treatments is not yet well-defined. Thus, this study aimed to evaluate the results of elective and simultaneous EVAR and LC.

**Methods:** Thirteen patients (mean age, 72 years) with concomitant large and asymptomatic AAA and asymptomatic cholelithiasis underwent simultaneous EVAR and LC.

**Results:** Post-operative mortality was absent, and the morbidity rate was 7%. The mean total duration of the procedure was 142 min. The mean duration of fluoroscopy was 19 min, and the mean radiation dose was 65 mGy. The mean amount of iodinated contrast injected was 49 mL. The timing of oral fluid intake was 28 h (range, 24–48 h) and that of the oral low-fat diet was 53 h (range, 48–72 h). No patient presented with an aortic graft infection during the entire follow-up period (mean duration, 41 months). The mean length of post-operative hospital stay was 6 days (range, 5–8 days). Late survival was 85%, and the exclusion of AAA was 100%.

**Conclusion:** Simultaneous EVAR and LC can be performed safely, allowing effective and durable treatment under both AAA and cholelithiasis conditions.

## Introduction

The incidence of asymptomatic concomitant cholelithiasis and abdominal aortic aneurysm (AAA) is ~6%, and the incidence of post-operative acute cholecystitis in patients undergoing isolated repair of AAA is 18% (1–3). These data apply to open aneurysm repair and justify the simultaneous treatment of both conditions through laparotomy, with cholecystectomy being performed once aneurysm repair is completed and the aortic graft is covered with peritoneum to treat both conditions while minimizing the risk of intraoperative contamination of the aortic graft (2). With the increasing use of endovascular aneurysm repair (EVAR) and the availability of laparoscopic cholecystectomy (LC) for treating AAA and cholelithiasis, respectively, this paradigm has changed. Isolated cases of simultaneous open retroperitoneal aneurysm repair combined with LC (4) and EVAR combined with LC (1, 5) have been reported. The actual incidence of cholecystitis complicating the post-operative course of EVAR in patients with asymptomatic lithiasis of the gallbladder associated with an AAA undergoing EVAR is not well-known and is probably a rare event. Nonetheless, it bears a definite risk of graft infection and may seriously complicate the course of an otherwise simple and minimally invasive procedure such as EVAR. Thus, we prospectively performed simultaneous LC in patients undergoing EVAR for a large asymptomatic AAA with an asymptomatic lithiasis of the gallbladder. To test the validity of this treatment, we retrospectively reviewed the results of a preliminary and small series of patients treated with this simultaneous approach.

## Patients and Methods

From January 2013 to July 2020, 13 consecutive patients [11 men; mean age, 72 years (range, 59–82 years)] underwent simultaneous EVAR followed by LC for an AAA associated with cholelithiasis at an academic tertiary care hospital and an affiliated surgical center. All patients provided written consent for the combined procedures, whereas institutional ethics committee approval was waived due to the retrospective nature of the study.

Both conditions were not complicated and asymptomatic at the time of hospital admission for treatment, and all AAAs were standard and infrarenal with a neck of at least 17 mm in length. Demographics and risk factors are shown in Table 1.

**Table 1:** Demographics, risk factors and baseline characteristics.

**Variable**	**Measure**
Age (years)	59 (range, 59–82)
Male gender (*N*, %)	11, 85
HTA (*N*, %)	10, 77
Current smokers (*N*, %)	7, 54
Dyslipidemia (*N*, %)	5, 38
Diabetes (*N*, %)	4, 31
CAD (*N*, %)	3, 23
CRI (*N*, %)	1, 8
PAD (*N*, %)	1, 8
ASA II (*N*, %)	9, 70
ASA III (*N*, %)	2, 15
ASA IV (*N*, %)	2, 15
BMI (kg/m^2^)	28, 8 (range, 23.2–34.0)
Length of AAA neck (mm)	21 (±4)
Diameter of AAA neck (mm)	24 (±3)
Neck circumference calcification (%)	8 (±2)
Diameter of common iliac arteries (mm)	9 (±2)
Fasting gallbladder volume (mL)	31.4 (±12.3)

*AAA, abdominal aortic aneurysm); ASA, American Society of Anesthesiologists; BMI, Body Mass Index; CAD, Coronary Artery Disease; CRI, Chronic Renal Insufficiency (Serum Creatinine >120 mmol/L); HTA, Arterial hypertension; PAD, Peripheral Artery Disease*.

Preoperative workup included the ultrasound analysis of the abdomen, magnetic resonance imaging of the gallbladder and biliary tract, duplex ultrasound analysis of the supraaortic trunk, and angio–CT scan of the thoraco-abdominal aorta and lower limbs. Two patients underwent coronary angiography followed by percutaneous intervention (PCI) for the treatment of significant, yet asymptomatic stenoses of the coronary arteries. All patients were operated under an oral statin regimen (atorvastatin, 20 mg/day), started at least a week before the operation. In addition, seven patients (74%) were operated under a single antiplatelet treatment (acetylsalicylic acid, 100 mg/day), whereas two patients (15%) undergoing previous PCI were operated under dual antiplatelet treatment (acetylsalicylic acid, 100 mg/day + clopidogrel 75 mg/day).

**Figure 1:**
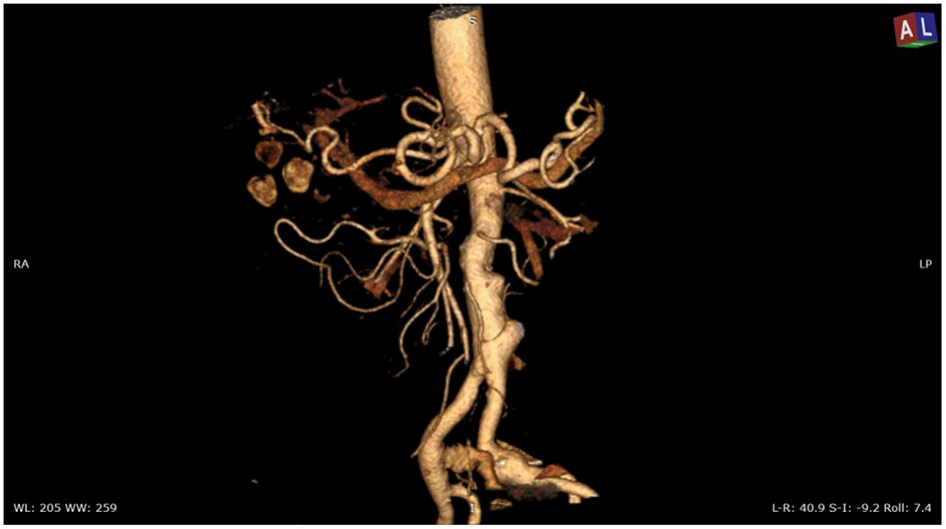
Angio-CT scan of the thoracoabdominal aorta showing aneurysmal degeneration of penetrating aortic ulcers in the infrarenal aorta associated with lithiasis of the gallbladder.

**Figure 2:**
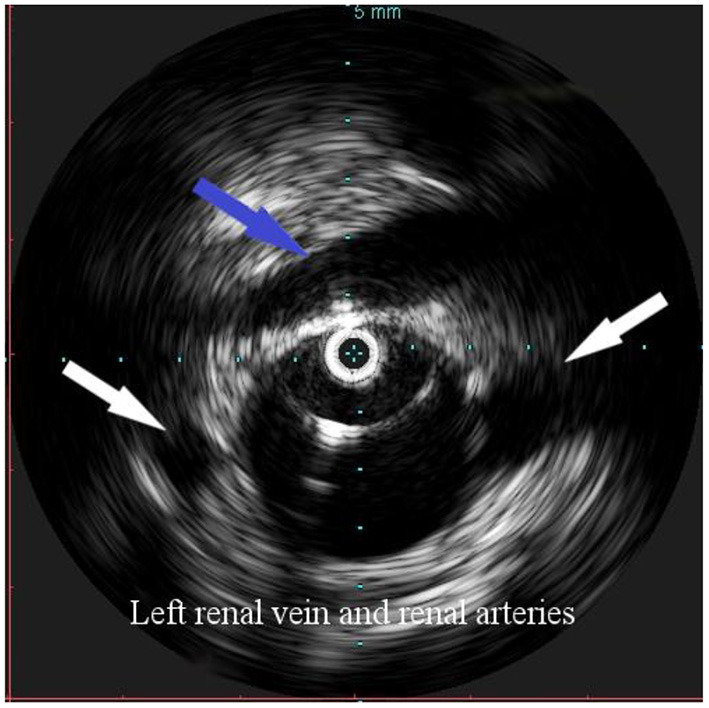
Intravascular ultrasound (IVUS) identification of the ostia of renal arteries with the left one being lower (white arrows), in order to define the proximal landing zone of the graft. The left renal vein can be seen crossing the anterior aortic wall (arrow).

**Figure 3:**
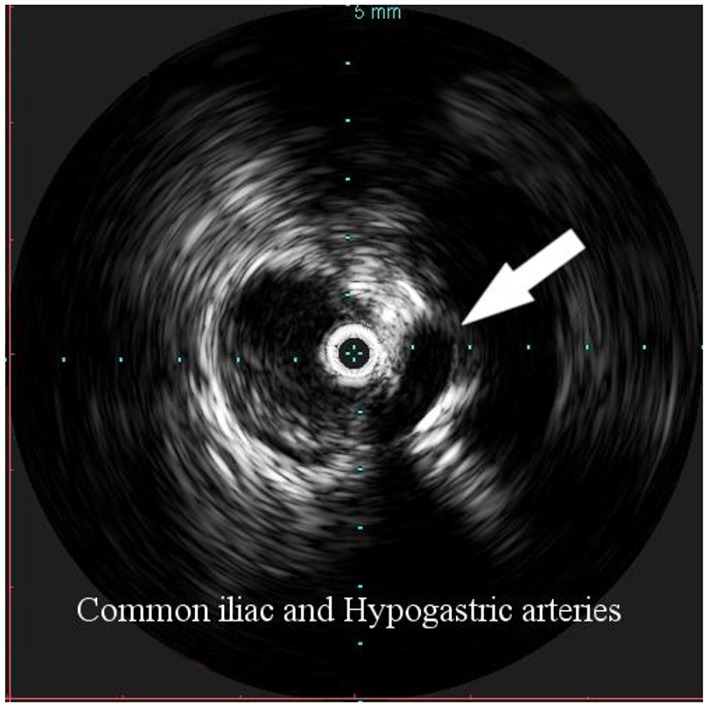
Intravascular ultrasound (IVUS) identification of the hypogastric artery offspring (arrow), in order to identify the distal landing zone of the graft.

Overall, the mean greater diameter of the AAA was 60 mm (range, 40–72 mm); all patients but one had an AAA of at least 55 mm in diameter. The latter presented with an aneurysmal degeneration of a double-penetrating ulcer of the infrarenal aorta with a significant bleb, raising concern for a possible impending rupture (Figure 1). Thus, a pre-operative leukocyte scintigram of the patient was also obtained to rule out a possible septic etiology of the bleb itself, eventually resulting in bacterial seeding during a subclinical episode of bacteremia arising from the gallbladder. The combined procedures were performed under general anesthesia in an operating room equipped with two types of C-arm: General Electric OEC 98000 Plus (General Electric Medical Systems Inc., Salt Lake City, UT, USA) and General Electric Innova 4100 (General Electric Medical Systems Inc., Salt Lake City, UT, USA). The radiation dose absorbed was measured using a Diamentor M4–KDK detector (PTW, Freiburg, Germany). EVAR was performed first in all cases. Arterial access was achieved through femoral artery cutdown in four (31%) patients and percutaneously in the remaining 9 (69%) patients. The percutaneous access was closed with a single Perclose Proglide SMC device (Abbott Vascular, Santa Clara, CA) for sheaths with a diameter of 11 F or less and with two devices for sheaths with diameters exceeding 11 F. In the case of femoral artery cutdown, both EVAR and LC were performed under the same general anesthesia; in the case of percutaneous femoral access, EVAR and LC were performed under local and general anesthesia, respectively. All EVAR were assisted by intravascular ultrasound (IVUS) to minimize and possibly limit the use of iodinated contrast medium for completion angiography, which is required at the end of the procedure to rule out a type I endoleak; the 0.35 IVUS probe does not incorporate a chromoflow Doppler probe, which could detect type I endoleak. The technique has been previously described in extent (6): briefly, after systemic heparinization (5000 IU of heparin sodium intravenously) and sheath introduction, the IVUS probe (Volcano Vision PV 8.2 F, Volcano Japan Inc., Tokyo, Japan) was advanced up to the diaphragm and pulled back to locate the ostia of the renal arteries (Figure 2), which were marked on the fluoroscopic monitor, considering the identification of the lower renal artery, in case of different offspring levels between the two. The main body of the endograft (Bolton Treo, Bolton Medical, Sunrise, FL, USA) was deployed. The IVUS probe was used to locate the ostia of the hypogastric arteries (Figure 3), to reach the gate, and to assess the optimal distal landing zone. Completion angiography and closure of the femoral access completed the procedure.

After protamine reversal of half of the heparin dose (2,500 IU), LC was performed using three trocars. The gallbladder was removed through the umbilical port using a collection bag and was systematically sent for bacterial culture. An infrahepatic drain was left in place for 48 h. Pre-operative antibiotic treatment consisted of 2 g of intravenous cephazolin, which was administered for 48 h, and subsequently replaced with 1 g of oral amoxicillin + clavulanic acid every 8 h for the next 10 days. All patients underwent a control abdominal ultrasound study before discharge from the hospital and a control angio-CT scan of the thoraco-abdominal aorta 1 month after the procedure. Subsequent controls consisted of a duplex ultrasound study of the abdomen every 6 months for the first year and every 12 months thereafter, coupled with a CT scan of the thoraco-abdominal aorta every 24 months. The mean follow-up duration was 41 months (range, 4–93 months).

The endpoints of the study were post-operative mortality and morbidity, total duration of the procedure, EVAR and LC, mean duration of fluoroscopy, mean amount of iodinated contrast injected, mean radiation dose, timing of oral fluid and solid food administration, occurrence of graft infection, duration of post-operative stay in the hospital, late survival, and late exclusion of the AAA. The radiation dose was defined as the kinetic energy released per unit of matter (KERMA) and expressed as mGy.

## Results

The mean length and mean diameter of the aneurysmal neck was 21 mm (±4 mm) and 24 mm (±3 mm), respectively, the mean diameter of the common iliac arteries was 9 mm (±2 mm), and the mean percentage of neck circumference calcification was 8% (±2%). The fasting gallbladder volume was 31.4 mL (±12.3 mL).

No patient died post-operatively. One patient required surgical exposure and hemostasis of the common femoral artery due to malfunctioning of the Perclose device. No other access site complications such as bleeding, infection, or thrombosis, respiratory complications, or cardiac complications were observed. Therefore, the overall incidence of post-operative morbidity is 7%.

The mean total duration of the procedure was 142 min (range, 115–180 min), whereas the durations of EVAR and LC were 103 min (range, 90–140 min) and 38 min (range, 25–60 min), respectively. The mean duration of fluoroscopy was 19 min (range, 10–35 min), and the mean radiation dose was 65 mGy (range, 50–100 mGy). The mean amount of iodinated contrast injected was 49 mL (range, 40–80 mL). The timing of oral fluid intake was 28 h (range, 24–48 h) and that of the oral low-fat diet was 53 h (range, 48–72 h). No patient presented with an infection of the aortic graft either post-operatively or during the entire follow-up period. The mean duration of post-operative hospital stay was 6 days (range, 5–8 days).

**Figure 4:**
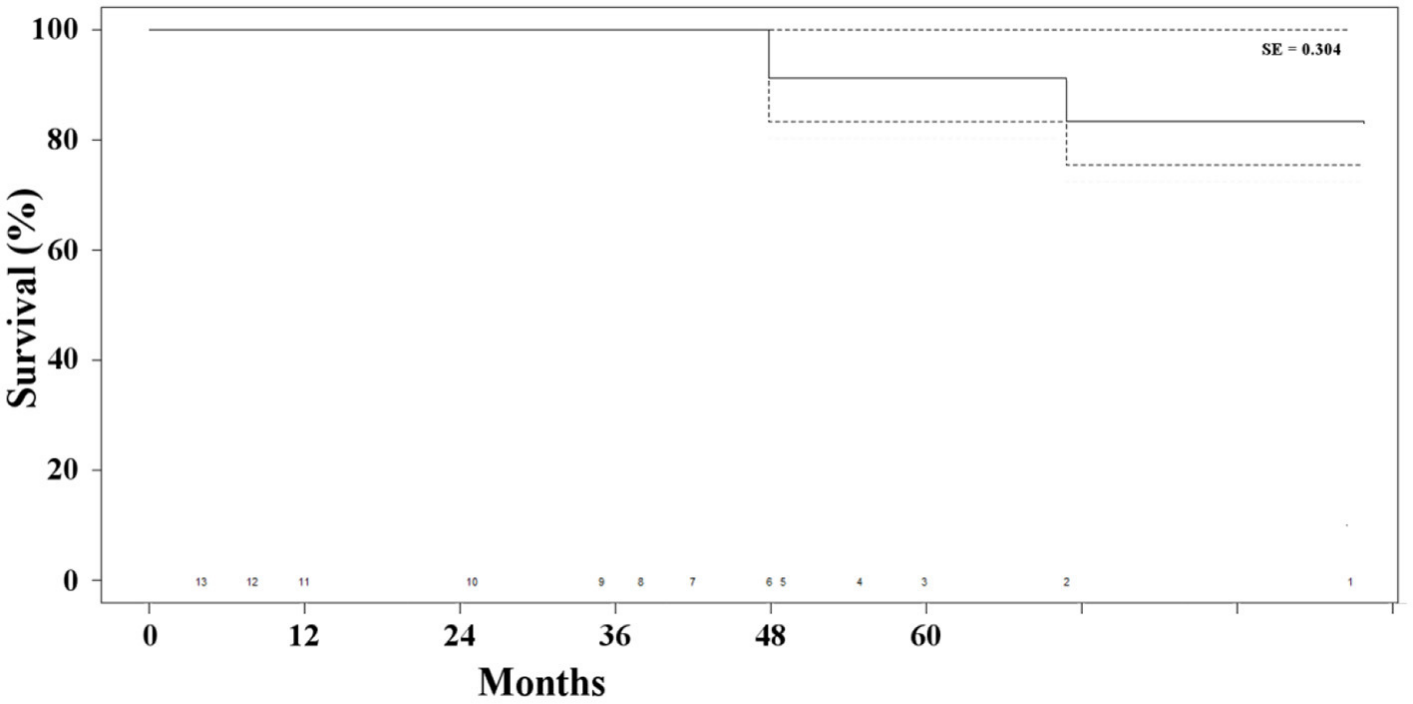
Kaplan-Meier estimate of survival of the patients' population.

Two patients (15%) died of unrelated causes (i.e., cancer and stroke) at 48 and 71 months, respectively, after the combined procedure. The overall survival rate was 85% at 41 months (Figure 4). None of the patients presented any sign of type I endoleak or biliary-related complications. Three patients (23%) presented with persistent type II endoleak, which was not associated with the increase in the diameter of the aneurysm and did not require any additional treatment. Overall, freedom from any biliary or aneurysm-related reintervention was 100% (Table 2).

**Table 2:** Endpoints of the study.

**Variable**	**Measure**
Post-operative mortality	–
Post-operative morbidity (*N*, %)	1, 7
Mean total lenght of the procedure (min.)	142 (range, 115–180)
Mean lenght of EVAR (min.)	103 (90–140)
Mean lenght of LC (min.)	38 (range, 25–60)
Mean duration of fluoroscopy (min.)	19 (range, 10–35)
Radiation dose (mGy)	65 (range, 50–100)
Mean amount of iodinated contrast (ml)	49 (range, 40–80)
Timing of oral fluid intake (hours)	28 (range, 24–48)
Timing of oral low-fat diet administration (hours)	5 (range, 48–72)
Mean post-operative lenght of stay (days)	6 (range, 5–8)
Late survival (%)	85
Freedom from further treatment (%)	100

## Discussion

The findings of this study show that simultaneous EVAR and LC for the treatment of concomitant AAA and lithiasis of the gallbladder, respectively, can be safely performed whenever both conditions are asymptomatic and non-complicated. This treatment strategy eliminates the possibility of complications of either of the two untreated conditions in the case of separate treatment. Whenever a cholecystectomy or any other abdominal surgical procedure is performed first, a major concern has always been the possibility of enhancing the probability of rupture of an associated and untreated AAA due to an increase in post-operative collagenase activity (1–3, 7). On the other hand, prior treatment of AAA may expose the patient to post-operative cholecystitis, thus severely increasing the risk of infection of the aortic graft. During the open repair of an AAA, this dilemma is overwhelmed, and the issue is addressed by performing cholecystectomy through the same laparotomy once the repair of aneurysm and covering of the graft with peritoneum is completed. The rationale is that the addition of cholecystectomy at the end of aneurysmectomy does not significantly increase the complexity and duration of the operation (8).

However, with the widespread use of endovascular techniques for the treatment of even complex AAA and the declining indications for open aneurysm repair, the issue of timing of treatment of the two conditions remains. The probability of acute cholecystitis complicating the post-operative course of EVAR, in case an associated asymptomatic lithiasis of the gallbladder is estimated to be fairly low, thus allowing EVAR to be performed first, followed by cholecystectomy (9). Nonetheless, even in an elective setting and when dealing with both asymptomatic diseases, the possibility of combining two minimally invasive methods of treatment, EVAR and LC, during the same procedure seems appealing because it allows simultaneous and durable treatment of both conditions. This may be justified if it does not complicate the normal post-operative course of the two procedures. The results of this study seem to support this hypothesis, although limited to both conditions being asymmetrical. Regarding symptoms, the symptomatic condition deserves treatment priority with the best surgical access and any eventually associated treatment being chosen on a case-by-case basis. Simultaneous EVAR and LC after prior medical treatment for cholecystitis have already been reported (1).

Three major issues were of concern in this patient's series: the possibilities of major bleeding from the hepatic bed of the gallbladder, graft infection, and pneumoperitoneum–induced kinking or migration of the aortic endograft. Reversal of half dose of sodium heparin after completing EVAR avoided any major bleeding even in patients under dual antiplatelet regimen due to recent PCI, in agreement with other reports (10), and no post-operative transfusion of blood units was required in this series. Intraoperative antibiotic prophylaxis with cephalosporin, followed by oral antibiotic treatment for ten post-operative days, allowed the absence of any post-operative sepsis or graft infection. Approximately 3.7% of EVAR cases require subsequent later conversion, and of these, only 9.5% are due to infection, accounting for a fairly low incidence (11). Nonetheless, bacteremia from an apparently asymptomatic cholelithiasis is among the most frequent causes of late aortic graft infection of an apparently unknown cause, supporting the simultaneous treatment of both conditions on a cost-benefit analysis. Finally, no pneumoperitoneum-induced graft kinking, migration, or anomaly of any other kind was observed in this series compared to previous reports (12).

### Limitations

This study has three limitations: its retrospective nature, the small number of patients, and the lack of a control group. Nonetheless, data were objectively collected and analyzed, and because of its relative rarity, it is unlikely to foresee a prospective, randomized study on the analyzed setting with a short delay.

## Conclusion

Simultaneous EVAR and LC for the treatment of concomitant asymptomatic AAA and gallbladder lithiasis can be performed safely, allowing effective and durable treatment of both conditions.

## Data Availability Statement

The raw data supporting the conclusions of this article will be made available by the authors, without undue reservation.

## Ethics Statement

Ethical review and approval was not required for the study on human participants in accordance with the local legislation and institutional requirements. The ethics committee waived the requirement of written informed consent for participation.

## Author Contributions

GI, FC, and PU contributed to the conception and design of the study, analyzed and interpreted the data, and made critical revisions to the manuscript. RP, PN, and CF were responsible for literature search, data collection, and extraction of relevant information. GI wrote the manuscript. All authors contributed to the article and approved the submitted version of the manuscript.

## Conflict of Interest

The authors declare that the research was conducted in the absence of any commercial or financial relationships that could be construed as a potential conflict of interest.
